# The Effect of High Intensity Focused Ultrasound Keratoplasty on Rabbit Anterior Segment

**DOI:** 10.1155/2017/6067890

**Published:** 2017-02-09

**Authors:** Menglei Wang, Meixuan Li, Pisong Yan, Qiang Luo, Yu Zhang, Zhiyu Du

**Affiliations:** ^1^Department of Ophthalmology, The Second Affiliated Hospital, Chongqing Medical University, Chongqing 400010, China; ^2^Medal Eye Institute, Chongqing 400050, China; ^3^Key Laboratory of Molecular Biology for Infectious Diseases (Ministry of Education), Institute for Viral Hepatitis, Department of Infectious Diseases, The Second Affiliated Hospital, Chongqing Medical University, Chongqing 400010, China

## Abstract

*Purpose.* To evaluate the safety of high-intensity focused ultrasound keratoplasty as a treatment for presbyopia by examining its effect on the rabbit anterior segment.* Methods.* The right corneas of 36 New Zealand rabbits were treated with HIFU keratoplasty. The animals were sacrificed at 1, 7, 15, 30, 60, and 90 days after operation. Collagen type I, MMP-2, and MMP-9 were evaluated using immunohistochemistry. For the detection of apoptosis, the TUNEL method was applied. The SOD and MDA levels were analyzed with assay kits.* Results.* Collagen type I, MMP-2, and MMP-9 levels were altered after the operation but returned to normal within 90 days. The apoptotic index (AI) of the corneal cells decreased from 1 to 30 days gradually. No apoptosis was observed in the epithelial cells of the lens, and the SOD and MDA levels were normal at any time point.* Conclusion.* After HIFU keratoplasty, the histomorphology of the cornea changed, the corneal collagen type I levels decreased, the corneal MMP-2 and MMP-9 levels increased, and the corneal cells underwent apoptosis for a period of time. Ninety days after the operation, the levels returned to normal, and the lenses were not affected. Thus, HIFU presents good biological safety for eyes.

## 1. Introduction

Over the past decade, high-intensity focused ultrasound (HIFU), a potential noninvasive treatment, has been extensively used in the treatment of tumors. In ophthalmology, therapeutic ultrasound is only used in glaucoma [1] and ultrasonic drug delivery [2]. Its use for the treatment of choroidal melanoma is still in the research stage [3]. By increasing the temperature above 43–67°C within a few seconds, HIFU induces thermal lesions in the focal zone [3]. It is known that when the temperature of the corneal stroma collagen reaches 65–70°C, the collagen reaches a permanent state of contraction [4]. Thus, in 1990, HIFU was first proposed for inducing collagen shrinkage in the cornea [5]. The continuous development of HIFU has led to more advantages, such as smaller focal zones, better location accuracy, and more precise controllability. Because of its advantages, HIFU can be used to heat the peripheral cornea with great precision while keeping the neighboring tissue healthy. Therefore, we aimed to induce collagen shrinkage in the peripheral cornea by HIFU and to create a steeper transition between the focal zone and the untreated area to increase the corneal curvature.

The thermal effect of HIFU induces thermal lesions. Corneal injury involves superficial penetration of the epithelium and anterior part of the stroma, leading to tissue repair, which is often the onset of corneal fibrosis [6]. Myofibroblasts synthesize the extracellular matrix (ECM), and corneal collagen is the primary ECM component involved in the wound-healing response to corneal damage [7]. The corneal collagen reflects the stability of the cornea. The ECM is primarily processed by matrix metalloproteinases (MMPs), a family of zinc-dependent proteolytic enzymes involved in corneal inflammation, epithelial regeneration, stromal wound healing, and neovascularization [8]. MMPs are important indicator of corneal remolding. When a lens suffers an injury, increased oxidative stress causes lipid peroxidation, which results in increased malondialdehyde (MDA) levels. The oxidative stress results from the excessive production of free radicals or a reduced lenticular antioxidant defense. Superoxide dismutase (SOD) is the main antioxidant enzyme in the lens [9].

In previous research, we preliminarily established a new technique for correcting presbyopia and demonstrated that HIFU keratoplasty can be utilized to increase the rabbit corneal curvature [10]. In this study, we aimed to evaluate the safety of HIFU keratoplasty by investigating the changes in collagen type I, matrix metalloproteinase-2 (MMP-2), and matrix metalloproteinase-9 (MMP-9) in the cornea; the levels of SOD and MDA in the lens; and the apoptosis of corneal cells and lens epithelial cells after HIFU keratoplasty on the rabbit cornea.

## 2. Materials and Methods

### 2.1. Animals

Thirty-six healthy New Zealand White rabbits weighing 2000 to 2500 g, as recipient animals, were purchased from the Laboratory Animal Center of Chongqing Medical University. At the time of enrollment, no clinical signs of ocular surface disturbance were observed with a slit lamp. The corneas of the right eyes of the 36 rabbits were treated with HIFU keratoplasty, with the left eyes as the control group. Six animals were sacrificed at scheduled time points, 1 day, 7 days, 15 days, 30 days, 60 days, and 90 days, after HIFU keratoplasty. All of the procedures in this research study were approved by the Animal Care and Use Committee at Chongqing Medical University and conformed to the Association for Research in Vision and Ophthalmology statement for the Use of Animals in Ophthalmic and Vision Research.

### 2.2. HIFU Keratoplasty Procedure

The animals were anesthetized by giving them an intravenous injection of 30 mg/kg of pentobarbitone sodium (Pharmaceutical Co., China), and a local anesthetic containing 0.4% oxybuprocaine hydrochloride (Benoxil, Santen Pharmaceutical Co., Osaka, Japan) was applied to the corneas before the HIFU keratoplasty. The limbs of the rabbits were fixed on a fixed table. The fascia of the corneal limbus was pulled with surgical lines to maintain the cornea level. HIFU keratoplasty treatments were delivered with a prototype (Chongqing HIFU Technology Co., Ltd., Chongqing, China). Continuous therapeutic energy was emitted from a focal ultrasound transducer. The focal length was 5 mm, and the focal zone was 65 *μ*m. The working frequency was 10.2 MHz, and the therapy power was set at 1 W for an exposure time of 6 seconds. The ultrasound transducer was placed on a rotary motor with uniform velocity and oriented perpendicular to the corneal surface. The ultrasound transducer and the corneal surface did not have direct contact with each other, and the ultrasonic coupling agent acted as the medium. The focal length of the transducer was adjusted by a vernier caliper to focus on the same depth as the rabbit corneal stroma. The duration for one cycle of the rotary motor was also 6 seconds, and the diameter of the ring was 9 mm. Eye drops containing a steroid and antibiotics (dexamethasone/tobramycin, Alcon Laboratories Inc.) were administered to the eyes at the end of the treatment.

### 2.3. Tissue Extraction

Six animals were sacrificed at scheduled time points: 1 day, 7 days, 15 days, 30 days, 60 days, and 90 days, after HIFU keratoplasty using intravenous pentobarbital. The eyes were immediately enucleated, and three of them were fixed in 4% paraformaldehyde in 0.1 M phosphate-buffered saline for 24 hours and then transferred to 70% ethanol until processing with paraffin. The tissue samples were embedded in paraffin and sectioned to a thickness of 4 *μ*m. The sections were evaluated with hematoxylin-eosin staining, immunohistochemistry, and TUNEL. The other three lenses were extracted and washed with phosphate-buffered saline. After drying on filter paper, the lenses were ground into a homogenate while cooling using an ice bath. Finally, the homogenate was centrifuged at 1,600 ×g at 4°C for 10 min, and the supernatant was used for analysis.

### 2.4. Hematoxylin-Eosin Staining

Paraffin-embedded sections were deparaffinized, dehydrated, and stained with HE. The hematoxylin–eosin (HE) kit was purchased from Zhongshan Jinqiao Biotechnology.

### 2.5. Immunohistochemistry

Tissue sections were deparaffinized in xylene and gradually hydrated through graded alcohol. The sections were subjected to standard antigen retrieval in 0.1% trypsin for 30 min at 37°C and quenched with 3% hydrogen peroxide for 10 min at room temperature. The sections were then blocked with 10% normal goat serum for 20 min at room temperature prior to primary antibody incubation. Primary polyclonal antibodies of rabbit anti-rabbit collagen type I (1 : 20, Bioss Bio., China), rabbit anti-rabbit MMP-2 (1 : 50, Boster Bio., China), and rabbit anti-rabbit MMP-9 (1 : 20, Bioss Bio., China) were applied overnight at 4°C. After rinsing with 0.01 mol/L phosphate buffer solution (PBS) (pH 7.4), sections were incubated with biotinylated secondary antibody (goat anti-rabbit IgG, Zhongshan Jinqiao Bio., China) for 15 min at 37°C. After further washing with PBS, the sections were incubated with complex/horseradish peroxidase (Zhongshan Jinqiao Bio., China) for 15 min at 37°C and then with diaminobenzidine (Zhongshan Jinqiao Bio., China) for 10 min at room temperature. Slides were counterstained with hematoxylin before dehydration, vitrification, and mounting. Incubation with PBS, instead of the primary antibody, was performed as a control for background staining.

The intensity of positive staining for collagen type I, MMP-2, and MMP-9 was measured using Image-Proplus 6.0 software (Media Cybernetics, United States). All images were acquired using the same microscope (Olympus BX51, Olympus Corp., Japan) and camera (Olympus DP70, Olympus Corp., Japan) sets. Five consecutive nonoverlapping 400x high-power fields were selected. The average integrated optical density (IOD) and stained area per high-power field were calculated from these 5 fields. The intensity of positive staining in the tissue sections was analyzed based on the average optical density, which is measured as the mean integrated optical density (IOD) per stained area (*μ*m^2^) (IOD/area) for positive staining.

### 2.6. Staining with TUNEL

Tissue sections were deparaffinized in xylene and gradually hydrated through graded alcohol. For antigen retrieval, sections were incubated with 20 *μ*g/mL proteinase K (Boster Bio., China) at 37°C for 30 min and washed with PBS. Endogenous peroxidase activity was blocked by 3% hydrogen peroxide solution for 10 min at room temperature. Subsequently, the sections were incubated in a wet box at 37°C for 1 h with 50 *μ*L of reaction solution containing 5 *μ*L of TdT and 45 *μ*L of fluorescein-conjugated nucleotide mixed buffer (Roche, USA). For nuclear counterstaining, the cells were treated for 5 min with 4′,6-diamidino-2-phenylindole (DAPI) (Beyotime Bio., China) solution. A fluorescence microscope was employed for observation and imaging, and the sections were then washed with PBS. The immunological reaction was amplified in a wet box at 37°C for 30 min with POD (Roche, USA). Staining was performed using DAB and was observed using a microscope. After the nuclei were colored dark brown, the sections were stained by hematoxylin, dehydrated by ethanol, vitrificated by dimethylbenzene, and mounted by neutral resins. Five consecutive nonoverlapping 400x high-power fields were selected. The average count of total cell numbers and apoptotic cell numbers per high-power field were calculated from these 5 fields. The apoptotic index (AI) was determined as the number of apoptotic cells/total cells × 100%.

### 2.7. Determination of SOD Activity and MDA Levels

The SOD activity was determined using the Total SOD Assay Kit with WST-8 (Beyotime Bio., China), which is based on the ability of SOD to inhibit the oxidation of oxymine by O_2_^−^ produced from the xanthine–xanthineoxidase system. Following the manufacturer's protocol, the SOD activity was measured at a wavelength of 550 nm. MDA, a product of lipid peroxidation, was also analyzed by an assay kit (Beyotime Bio., China), following the manufacturer's protocol. MDA levels were measured at 532 nm for the formation of stable chromophoric with thiobarbituric acid (TBA).

### 2.8. Statistics

The Statistical Package for Social Sciences (SPSS) 23.0 software (SPSS Inc., Chicago, IL) was used for statistical analyses. Differences between the two groups at each time point were assessed with an independent *t*-test. For the analysis of results among six time points, the Kruskal-Wallis test was performed, followed by a post hoc comparison using the Nemenyi test. A two-tailed *P* < 0.05 was considered statistically significant.

## 3. Results

**Figure 1:**
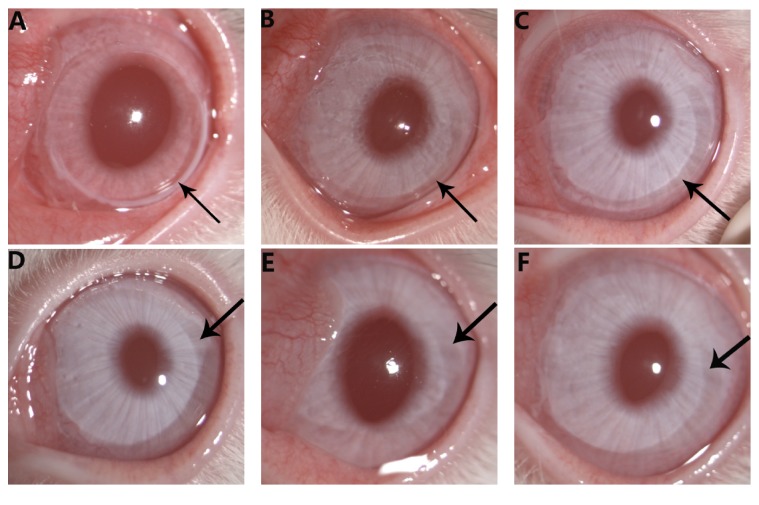
Corneal image acquired by a slit lamp microscope. (A) A white irradiated ring was observed in the peripheral cornea 1 day after operation (indicated by an arrow); ((B), (C), (D), (E), and (F)) the rings gradually faded from 7 to 90 days (indicated by an arrow).

During the study period, all corneas were stable without infection, perforation, or neovascularization, and the lenses remained transparent. A white irradiated ring was observed in the peripheral corneal stroma in the treated group 1 day after operation (Figure 1(A)). On day 7, the irradiated ring had faded significantly (Figure 1(B)). In the following observations, the white irradiated ring gradually faded away, with the cornea restored to transparent (Figures 1(C), 1(D), 1(E), and 1(F)).

**Figure 2:**
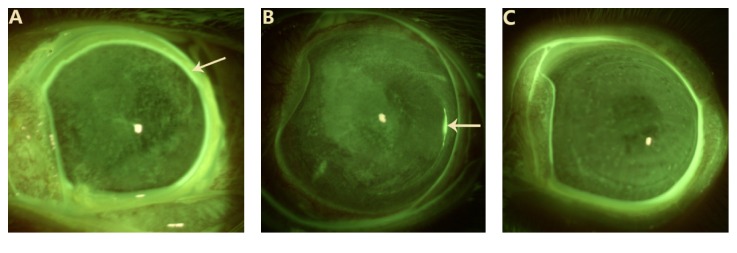
Corneal sodium fluorescein staining. (A) Corneal sodium fluorescein staining 1 day after the operation; part of the corneal epithelium was defective in the irradiated area (indicated by an arrow); (B) corneal sodium fluorescein staining 7 days after operation; a small amount of the corneal epithelium was defective in the irradiated area (indicated by an arrow); (C) 15 days after the operation, no sodium fluorescein staining was observed.

Corneal sodium fluorescein staining indicated that part of the corneal epithelium was defective in the irradiated area on day 1 (Figure 2(A)). On day 7, only a small amount of the corneal epithelium was defective in the irradiated area (Figure 2(B)). On day 15, the corneal epithelium was restored and intact (Figure 2(C)).

**Figure 3:**
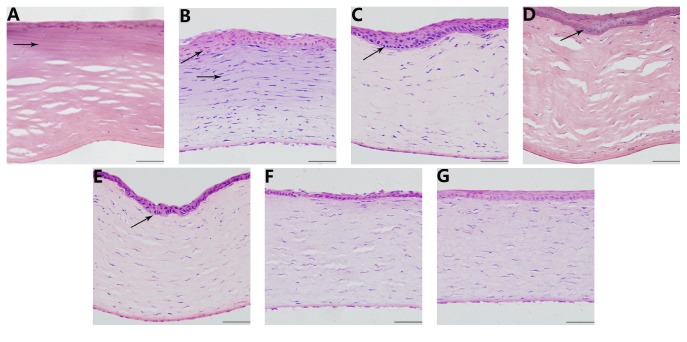
Hematoxylin-eosin staining of the cornea at different time points. (A) 1 day after the operation, the focal zone is deeply stained (indicated by an arrow), the fibers of the focal zone were swollen and fused, 400x; (B) 7 days after the operation, the focal zone showed a lighter staining, and the epithelium was hyperplastic (indicated by an arrow), 400x; ((C), (D), and (E)) 15 days, 30 days, and 60 days after operation, the focal zone disappeared and the epithelium was hyperplastic (indicated by an arrow). The fibers shrunk and became distorted, 400x; (F) 90 days after operation, epithelial hyperplasia was significantly reduced, and the matrix fibers were arranged in order, 400x; (G) hematoxylin-eosin staining of a cornea in the control group, 400x. Scale bar = 100 *μ*m.

HE staining indicated that the focal zone was deeply stained in the corneal stroma in the treated group 1 day after operation. The boundary of the focal zone was clear, and the fibers were swollen and fused. The epithelium and endothelium were intact, but the corneal stromal cells disappeared (Figure 3(A)). Seven days after the operation, the focal zone exhibited a lighter staining, and the fibers were still swollen and fused. The epithelium was hyperplastic, and the number of inflammatory cells increased (Figure 3(B)). From day 15 to 60, the focal zone disappeared. The hyperplasia of the epithelium gradually decreased with mild shrinkage of the matrix fibers (Figures 3(C), 3(D), and 3(E)). Ninety days after the operation, the epithelial hyperplasia was significantly reduced, and the matrix fibers were neatly arranged (Figure 3(F)). The histomorphology of the lenses was normal at each time point.

**Figure 4:**
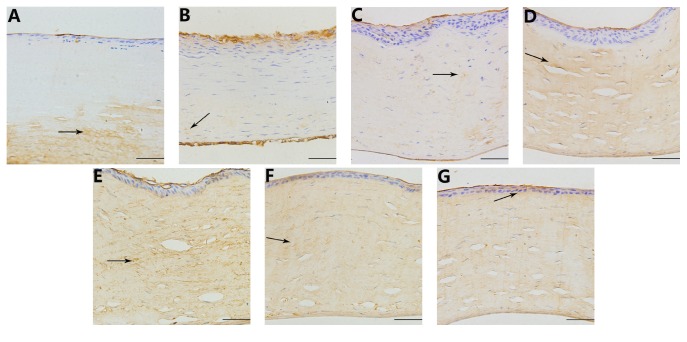
Immunohistochemistry of collagen type I in the cornea at different time points. ((A), (B)) 1 day and 7 days after operation, there was no expression of collagen type I in the focal zone. The expression of collagen type I out of the focal zone was normal (indicated by an arrow), 400x; (C) 15 days after the operation, the expression of collagen type I increased in the focal zone (indicated by an arrow), 400x; ((D), (E), and (F)) 30 days, 60 days, and 90 days after the operation, the expression of collagen type I was observed in the corneal epithelium, stroma, and endothelium (indicated by an arrow), 400x; (G) in the control group, collagen type I was expressed in the corneal epithelium, stroma, and endothelium, 400x. Scale bar = 100 *μ*m.

The average optical density (IOD/area) of collagen type I in the cornea was significantly lower compared with the control group at 1, 7, and 15 days after operation (*t* = 58.933, 28.070, 14.116, *P* < 0.05; Table 1). Collagen type I levels began to significantly decrease 1 and 7 days after the operation, with no significant difference between the two time points (*P* > 0.05; Figures 4(A) and 4(B)). Fifteen days after the operation, the levels began to increase (compared to day 7, *P* < 0.05; Figure 4(C)). On day 30, the expression of collagen type I returned to normal (Figures 4(D) and 7(a)).

**Figure 5:**
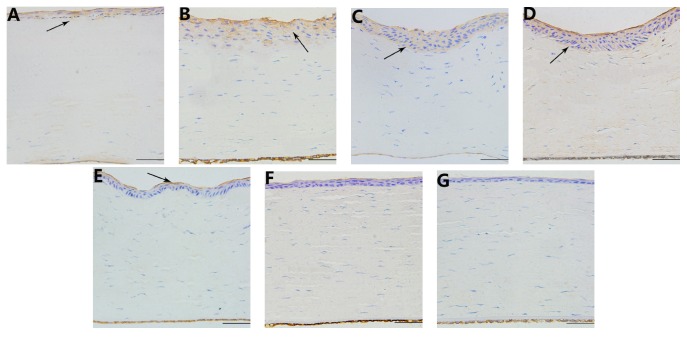
Immunohistochemistry of MMP-2 in the cornea at different time points. (A) On day 1, MMP-2 staining was found in the epithelium (indicated by an arrow), 400x; (B) on day 7, MMP-2 expression increased in the epithelium (indicated by an arrow). MMP-2 staining was also found in the boundary of the focal zone, 400x; ((C), (D)) on days 15 and 30, the expression of MMP-2 reached a maximum value, primarily in the epithelium (indicated by an arrow) but also in the stroma, 400x; (E) on day 60, the expression of MMP-2 was reduced, primarily in the epithelium (indicated by an arrow), 400x; (F) on day 90, there was no expression of MMP-2 in the epithelium or stoma, 400x; (G) there was no expression of MMP-2 in the epithelium or stoma in the control group, 400x. Scale bar = 100 *μ*m.

The average optical density (IOD/area) of MMP-2 in the cornea was significantly increased relative to the control group at 1, 7, 15, 30, and 60 days after operation (*t* = −19.14, −51.77, −90.54, −73.05, −29.86, *P* < 0.05; Table 1). MMP-2 levels began to increase on day 1 (Figure 5(A)) and increased progressively thereafter, reaching a maximum value on day 15 (compared to day 7, *P* < 0.05; Figure 5(C)) and remaining high until day 30 (compared to day 15, *P* > 0.05; Figure 5(D)). The levels then began to decline on day 60 (versus on day 30, *P* < 0.05; Figure 5(E)) and returned to normal on day 90 (Figures 5(F) and 7(b)).

**Figure 6:**
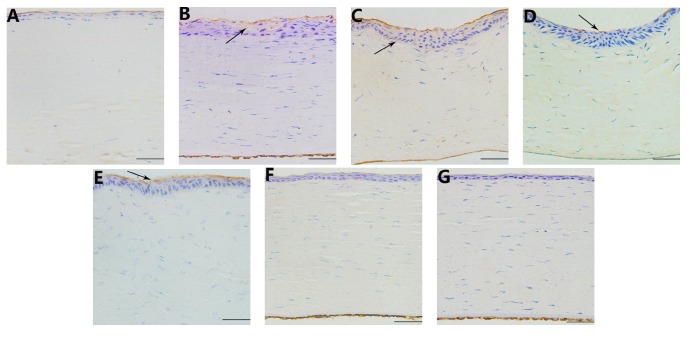
Immunohistochemistry of MMP-9 in the cornea at different time points. (A) On day 1, there was no expression of MMP-9 in the cornea, 400x; (B) on day 7, the expression of MMP-9 was observed in the epithelium (indicated by an arrow), 400x; (C) on day 15, the MMP-9 level peaked, primarily in the epithelium (indicated by an arrow) but also in the stroma, 400x; (D) on day 30, the expression of MMP-9 was reduced, with only a small amount in the epithelium and stroma (indicated by an arrow), 400x; (E) on day 60, MMP-9 showed low expression only in the epithelium (indicated by an arrow), 400x; (F) on day 90, there was no expression of MMP-9 in the epithelium or stoma, 400x; (G) no MMP-9 expression was observed in the epithelium or stoma in the control group, 400x. Scale bar = 100 *μ*m.

**Figure 7:**
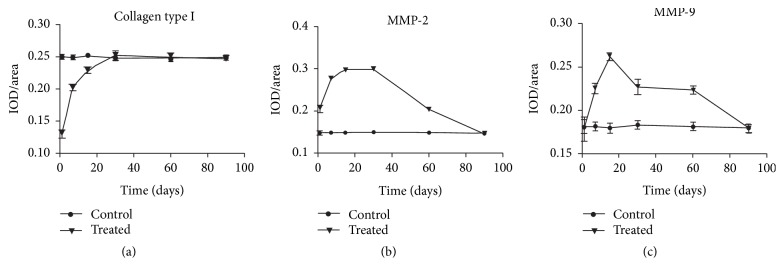
Changes in the average optical density (IOD/area). (a) The average optical density (IOD/area) of collagen type I in the cornea at different time points; (b) the average optical density (IOD/area) of MMP-2 in the cornea at different time points; (c) the average optical density (IOD/area) of MMP-9 in the cornea at different time points.

The average optical density (IOD/area) of MMP-9 in the cornea was significantly higher compared with the control group at 7, 15, 30, and 60 days after operation (*t* = −21.21, −43.85, −14.71, −22.97, *P* < 0.05; Table 1). The MMP-9 expression was increased at 7 days after operation (Figure 6(B)), and the peak value was reached on day 15 (versus day 7, *P* < 0.05; Figure 6(C)). The levels then began to decline on day 30 (versus day 15, *P* < 0.05; Figure 6(D)) until returning to normal on day 90 (Figures 6(F) and 7(c)).

**Figure 8:**
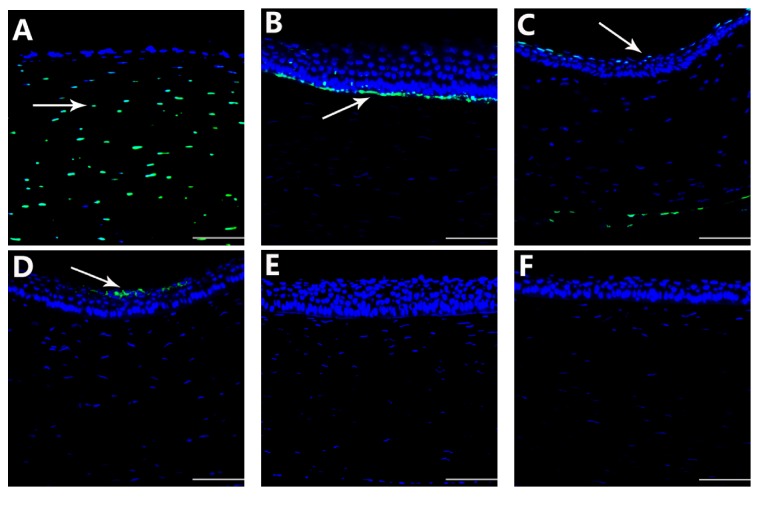
Apoptosis in the cornea at different time points observed by fluorescence microscopy. (A) On day 1, there was a large number of corneal apoptotic cells in the focal zone (indicated by an arrow), 400x; ((B), (C)) on days 7 and 15, the number of apoptotic cells decreased only in the presence of the epithelium and endothelium (indicated by an arrow), 400x; (D) on day 30, there were a few corneal apoptotic cells in the epithelium (indicated by an arrow) 400x; ((E), (F)) on days 60 and 90, there was no apoptosis in the cornea, 400x. Scale bar = 50 *μ*m.

The apoptotic index (AI) of the corneal cells began to increase and reached a maximum value on day 1 (Figure 8(A)). The index began to decline on day 7 (versus day 1, *P* < 0.05; Figure 8(B)) and remained similar until day 15 (versus day 7, *P* > 0.05; Figure 8(C)). On day 30, the index continued to decrease (versus day 15, *P* < 0.05; Figure 8(D)), returning to normal on day 60 (Figure 8(E), Table 2). No apoptosis was observed in the epithelial cells of the lens.

There were no significant differences in the SOD or MDA levels in the lens between the treated group and the control group at any time point (*P* > 0.05; Table 3).

## 4. Discussion

The use of heat to change the morphology of the cornea has been applied for different therapeutic objectives. The best known of these techniques is most likely conductive keratoplasty (CK). In the United States, CK has become a popular technique for the correction of low and moderate hyperopia due to its safety and effectiveness. However, refractive regression is a problem that should not be ignored. CK increases the local temperature to 65°C to change the refractive state through shrinkage of the stromal collagen [11]. Esquenazi et al. [12] found obvious thermal lesions in rabbit corneas, and the collagen fibers became distorted 2 weeks after CK, returning to normal 6 weeks after the operation. In this study, the focal zone appeared on day 1 and disappeared on day 15 in the cornea. The epithelium was hyperplastic, and the fibers shrunk and became distorted from 7 to 60 days after operation. The changes in the cornea were similar to those observed after CK, and the thermal effects of HIFU will also led to shrinkage of the collagen fibers; thus, the use of HIFU to correct presbyopia is feasible. Moreover, HIFU keratoplasty is a noninvasive treatment.

The corneal stroma primarily contains three types of collagen fibers: collagen type I, collagen type V, and collagen type VI [13]. Most of the collagen fibers are Type I collagen, accounting for approximately 60%. This type plays an important role in supporting the corneal structure and maintaining mechanical tension. A reduction in collagen type I levels will decrease the stability of the cornea [14]. Under physiological conditions, the metabolic rate of corneal collagen is low, in a relatively static state, and the collagenase is inactive. During the process of wound healing, collagen degradation due to collagenase is activated by IL-1, LPS, endotoxin, and other cytokines; then, fibroblasts resynthesize new collagen fibers [15]. HIFU keratoplasty produces a thermal lesion; then the epithelial cells, keratocytes, and inflammatory cells release a range of cytokines to stimulate epithelial regeneration and to deposit collagen. Thus, from 1 day to 15 days after operation, the collagenase was activated, and the expression of collagen type I decreased. From 30 days to 90 days after operation, the fibroblasts resynthesized new collagen fibers, and collagen type I returned to normal. Therefore, collagen type I levels in the cornea were not affected by HIFU keratoplasty, and the corneal structure remained stable.

Matrix-metalloproteinases (MMPs), specifically MMP-2 (gelatinase A) and MMP-9 (gelatinase B), have certain effects on wound healing [8], ocular surface disease [16], dry eye [17], and keratoconus [18]. MMPs are key effectors and regulators of inflammation, wound healing, tissue remodeling, and pathogenic processes [19]. MMP-2 and MMP-9 are mainly produced by epithelial cells and keratocytes, in an inactive form on the intact corneal epithelium and stroma [20]. After physical or chemical damage, inflammatory cells aggregate to the damaged area and synthesize or activate MMPs. At the same time, the keratocytes proliferate and migrate to the wound area and then differentiate into myofibroblasts to synthesize extracellular matrix (ECM) components, such as collagen type I, collagen type III, collagen type V, chondroitin sulfate, and proteoglycan. However, the structure of the newly synthesized ECM is disordered, and remodeling by activated MMPs is required. Excessive expression of MMPs may lead to haze and visual damage [21]. In our study, MMP-2 levels began to increase on day 1, reached a maximum value on days 15 and 30, and then began to decline on day 60 until returning to normal on day 90. MMP-9 levels began to increase on day 7, and a peak value was reached on day 15 and then began to decline on day 30 until returning to normal on day 90. On day 30, although the expression of collagen type I returned to normal, the collagen fibers were disordered. At this time, the strong expression of MMP-2 and MMP-9 had broken down the distorted collagen fibers and rebuilt the ECM structure. As the expression of MMP-2 and MMP-9 decreased, the arrangement of collagen fibers gradually became more orderly. Mulholland et al. [22] found that the expression of MMP-2 and MMP-9 were detected in rabbit corneas 4, 8, and 24 hours and 3, 7, and 14 days after lamellar keratectomy, with a peak value on day 7. Another study reported that the expression of MMP-2 appeared 1 week after corneal injury, when fibroblast proliferation was significantly increased, and then gradually decreased during the subsequent 7 months. The expression of MMP-9 was observed 2 days after corneal injury and disappeared within 2–4 weeks [23]. The time points for MMP-2 and MMP-9 activation and the duration of their expression have differed in various studies, which may be related to the type and severity of the injury. The changes in MMP-2 and MMP-9 levels after HIFU keratoplasty reflect the physiological process of corneal wound healing, without excessive expression.

Apoptosis is a kind of death process that allows for better adaptation to a changing environment and for the maintenance of homeostasis. Apoptosis is controlled by genes. At the present, HIFU has been widely used in the treatment of tumors by causing necrosis and apoptosis of tumor cells. In the focal zone of HIFU, the morphology changes, including karyopyknosis, and chromatin fracture fragments, which are surrounded by a cell membrane with some organelles such as mitochondria, then form apoptotic bodies and activate the caspase to regulate the process of apoptosis [24]. The apoptosis of corneal cells was observed within 1 month after HIFU keratoplasty. However, in other studies, within 24 hours after small incision lenticule extraction (SMILE) and femtosecond laser LASIK, within 4 days after Epi-LASIK and Flap-free Epi-LASIK, apoptosis of the corneal cells was observed [25, 26]. There were only a few apoptotic cells in the cornea at 7 days after operation, and the cells disappeared within 2 months. Thus, it is suggested that HIFU can be safely applied to the cornea.

It is easy for a cataract to form once the lens is damaged. Most researchers generally believe that although cataracts are regulated by a series of complex mechanisms, oxidative damage by free radicals is an important factor for their formation [27]. Superoxide dismutase (SOD) is an enzymatic antioxidant that acts by quenching O_2_ and converting it into H_2_O_2_ to protect the cell membrane from damage caused by the reactive oxygen species (ROS). Decreased SOD levels may lead to increased lipid peroxidation, resulting in cellular rigidity and deformability [28]. The lipid peroxidation represents oxidative damage, which is caused by superoxide anions, hydrogen peroxide, and hydroxyl radicals, resulting in a structural alteration of the membrane. Malondialdehyde (MDA) is the major end product of lipid peroxidation [29]. Numerous studies have reported that when a cataract forms, the activity of SOD decreases and the content of MDA increases [30]. In addition, oxidative damage can also cause apoptosis of the lens epithelial cells, which is another important factor in the formation of cataracts [31]. After HIFU keratoplasty, no significant differences were found in the levels of SOD and MDA between the treated group and the control group at any time point, and apoptosis of the lens epithelial cells was not observed. During the experiment, the lens remained transparent. The histomorphology of the lens in the experimental group showed no difference compared to the control group. Thus, the lens was not affected by HIFU keratoplasty.

## 5. Conclusion

After HIFU keratoplasty, the histomorphology of the cornea changed, the corneal collagen type I levels decreased, the corneal MMP-2 and MMP-9 levels increased, and the corneal cells underwent apoptosis for a period of time and returned to normal 90 days after operation. The lenses were not affected by HIFU keratoplasty. Therefore, HIFU is safe for both the cornea and the lens, and its application to presbyopia is feasible. However, the energy and positioning accuracy of HIFU should be further investigated.

## Figures and Tables

**Table 1:** The average optical density of collagen type I, MMP2, and MMP9 in the cornea for the two groups at six time points (IOD/area).

Time point	Collagen type I		MMP2		MMP9	
	Control group	Treated group	Control group	Treated group	Control group	Treated group
1 d	0.250 ± 0.004	0.130 ± 0.006^*∗*^	0.148 ± 0.006	0.205 ± 0.009^*∗*^	0.181 ± 0.008	0.178 ± 0.014
7 d	0.249 ± 0.004	0.202 ± 0.005^*∗*^	0.149 ± 0.004	0.277 ± 0.007^*∗*^	0.181 ± 0.005	0.225 ± 0.006^*∗*^
15 d	0.252 ± 0.003	0.229 ± 0.005^*∗*^	0.149 ± 0.003	0.298 ± 0.005^*∗*^	0.179 ± 0.006	0.262 ± 0.005^*∗*^
30 d	0.248 ± 0.005	0.252 ± 0.007	0.150 ± 0.005	0.299 ± 0.006^*∗*^	0.183 ± 0.005	0.227 ± 0.009^*∗*^
60 d	0.249 ± 0.006	0.250 ± 0.006	0.149 ± 0.005	0.204 ± 0.005^*∗*^	0.181 ± 0.005	0.223 ± 0.005^*∗*^
90 d	0.249 ± 0.004	0.247 ± 0.005	0.147 ± 0.004	0.146 ± 0.005	0.179 ± 0.006	0.179 ± 0.005

*P* ^∧^	—	<0.05	—	<0.05	—	<0.05

Data presented as the mean ± SD.

^*∗*^ *P* < 0.05 versus control group by independent *t*-test.

^∧^ *P* value represents a comparison among different time points, analyzed by the Kruskal-Wallis test.

**Table 2:** The percentage of apoptosis cells in the cornea at each time point, determined by TUNEL.

Time point	AI (%)
1 d	78.06 ± 1.94^bcd^
7 d	16.64 ± 0.77^ad^
15 d	13.34 ± 0.63^ad^
30 d	6.69 ± 0.33^abc^
60 d	—
90 d	—

Data presented as the mean ± SD.

^a^ *P* < 0.05 versus 1 d group, ^b^*P* < 0.05 versus 7 d group, ^c^*P* < 0.05 versus 15 d group, ^d^*P* < 0.05 versus 30 d group by the Kruskal-Wallis test followed by the Nemenyi test.

**Table 3:** SOD and MDA content in the lens for the two groups at six time points.

Time point	SOD		MDA	
	Control group	Treated group	Control group	Treated group
1 d	26.327 ± 5.474	27.887 ± 5.716	1.234 ± 0.449	1.219 ± 0.343
7 d	26.592 ± 3.758	26.512 ± 4.584	1.122 ± 0.474	1.074 ± 0.472
15 d	28.144 ± 4.893	26.937 ± 4.730	1.156 ± 0.306	1.216 ± 0.379
30 d	26.819 ± 5.766	26.252 ± 4.438	1.244 ± 0.528	1.206 ± 0.488
60 d	24.767 ± 3.559	24.042 ± 3.408	1.076 ± 0.342	1.024 ± 0.269
90 d	25.813 ± 2.826	23.946 ± 3.614	1.068 ± 0.332	1.061 ± 0.285

Data presented as the mean ± SD.
